# Three polymorphisms of renin-angiotensin system and preeclampsia risk

**DOI:** 10.1007/s10815-020-01971-8

**Published:** 2020-11-23

**Authors:** Chen Wang, Xiao Zhou, Huai Liu, Shuhui Huang

**Affiliations:** Department of Gynecology, Maternal and Child Health Affiliated Hospital of Nanchang University, Nanchang City, Jiangxi People’s Republic of China

**Keywords:** Polymorphism, AGT T704C, ACE I/D, AT1R A1166C, preeclampsia, risk

## Abstract

**Purpose:**

Some data suggest an association between the single nucleotide polymorphisms AGT T704C, ACE I/D, and AT1R A1166C and preeclampsia, but overall, the data are conflicting; the aim of our study was to discover a more stable and reliable association between these polymorphisms and PE risk.

**Methods:**

A comprehensive literature search for this meta-analysis was conducted. Odds ratios (OR) and 95% confidence intervals (CIs) were calculated to evaluate the strength, and heterogeneity test was conducted. Trial sequential analysis was also performed.

**Results:**

A total of forty studies were finally included in our meta-analysis. The AGT T704C polymorphism was associated with PE risk in three genetic models (dominant OR = 1.33, 95%CI = 1.12–1.59; heterozygote OR = 1.26, 95%CI = 1.05–1.52; homozygote OR = 1.44, 95%CI = 1.14–1.83). No heterogeneity was observed in the three genetic models for the ACE I/D polymorphism. For subgroup analysis by geography, no significant association was detected. Significant associations were observed in mixed race, early-onset, late-onset, and more than 200 subgroups for the AT1R A1166C polymorphism; however, only one study was analyzed in these subgroups.

**Conclusions:**

Our results indicated the AGT T704C and ACE I/D polymorphisms were associated with an increased risk of PE. Increased risks were also observed for the two polymorphisms in subgroups including Asians, Europeans, Caucasoid, and Mongoloid. Moreover, an increased PE risk with the ACE I/D polymorphism in the severe PE population was also detected. Regarding the AT1R A1166C polymorphism, weak associations were observed, but further studies are required.

**Electronic supplementary material:**

The online version of this article (10.1007/s10815-020-01971-8) contains supplementary material, which is available to authorized users.

## Introduction

Preeclampsia (PE) is a common complication of pregnancy characterized by hypertension and proteinuria after 20 weeks of gestation [1]; it is one of major causes of maternal-fetal and neonatal morbidity and mortality worldwide [2]. Knowing the risk factors for preeclampsia is critical for its prevention and treatment. Genetic factors play an important role in the genesis and development of PE and the genetic susceptibility to preeclampsia has generated great attention; the T allele of AGT may play a role in the pathogenesis of PE reported by Aung et al. [3],which indicated the gene polymorphisms in the renin-angiotensin-aldosterone system (RAAS) may be risk factors to PE.

During normal pregnancy, the upregulation of renin and aldosterone triggered by the stimulation of the RAAS system maintains the balance of blood volume and blood pressure [4]; however, for PE subjects, depression of the RAAS system with increased vascular resistance was observed, suggesting its crucial role in the pathogenesis of PE [5]. Angiotensin (AGT), angiotensin converting enzyme (ACE), and angiotensin II type 1 receptor (AT1R) are the three pivotal nodes in the RAAS system. The cleavage of AGT by renin contributes to the generation of angiotensin I, then ACE catalyzes the conversion of angiotensin I to a physiologically active angiotensin II. Finally, by binding to AT1R, angiotensin II regulates blood pressure by controlling sodium excretion [6]. Therefore, studies regarding the associations between single nucleotide polymorphisms in RAAS genes and PE risk are essential.

Associations between the polymorphisms of AGT T704C (the substitution of C to T at exon 2), ACE I/D (the insertion or deletion of an Alu 289 base pair sequence at intron 16), and AT1R A1166C (the change from C to A at 3’UTR) have been widely studied with conflicting results. To our best knowledge, differences in the geographic regions, ethnicity, and sample size could be reasons for the inconsistency. Moreover, the number of gestational weeks and the severity of PE have been reported to be associated with RAAS susceptibility gene polymorphisms [7–9], but these were not discussed in previous meta-analyses. Therefore, we conducted a comprehensive meta-analysis with trial sequential analysis to investigate the associations between the polymorphisms AGT T704C, ACE I/D, AT1R A1166C, and PE risk.

## Methods

### Literature search

**Fig. 1:**
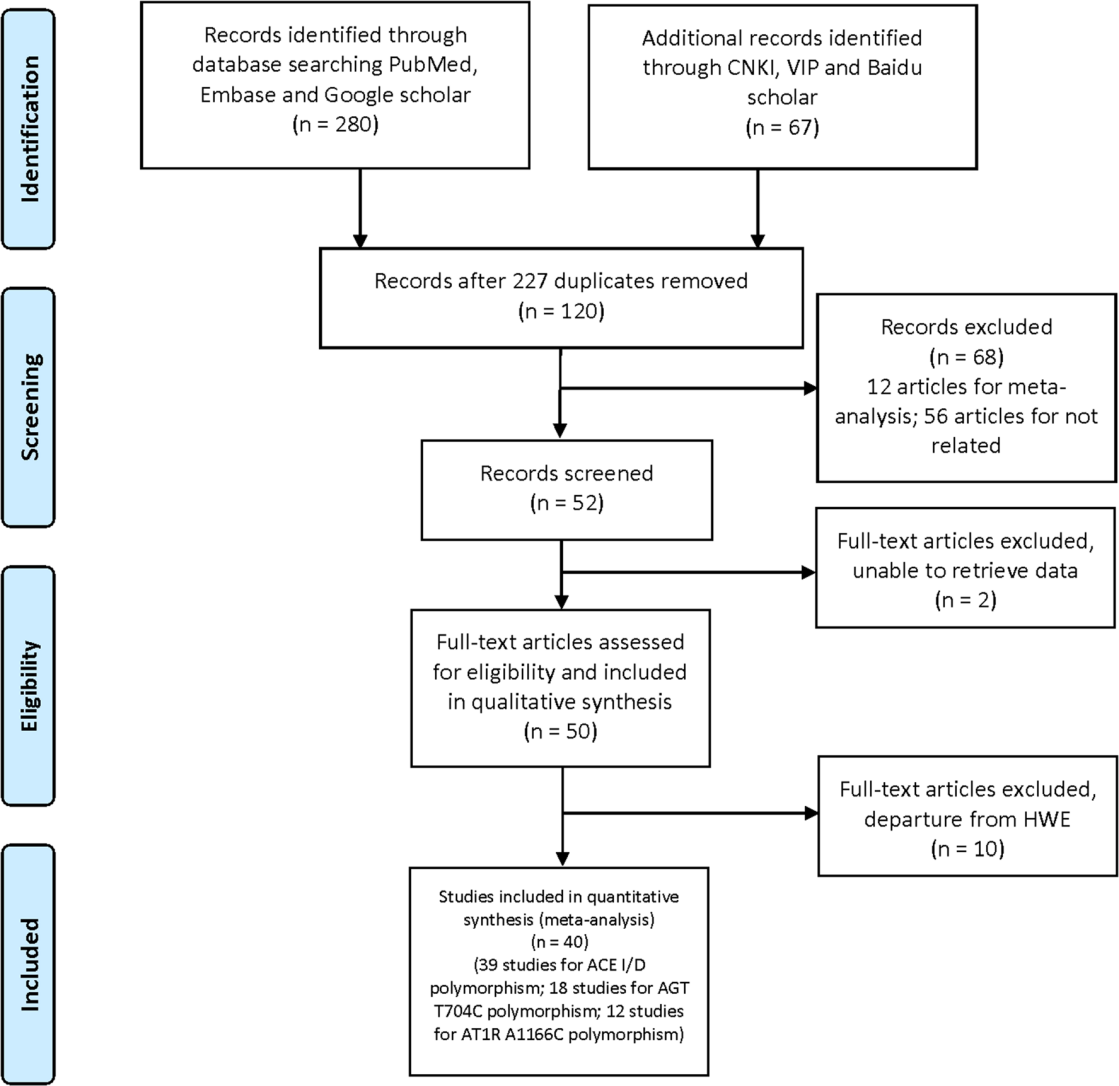
PRISMA 2009 flow diagram

PubMed, Embase, Google scholar, China National Knowledge Internet (CNKI), Baidu Scholar, Wan Fang, and VIP databases were comprehensively searched for studies regarding the associations between ACE insertion/deletion, AGT T704, and AT1R A1166C polymorphisms and preeclampsia susceptibility up to May 13, 2018. No language limitation was set. The following key words were used to discover relevant articles: “angiotensin-converting enzyme,” “angiotensin,” “angiotensin II type 1 receptor,” “ACE,” “AGT,” “AT1R,” “polymorphism,” “variant,” “single nucleotide polymorphism,” “SNP,” “preeclampsia,” “PE,” “hypertension,” and “pregnancy-induced hypertension syndrome.” The references of relevant studies were also screened by hand to identify potential studies. Our work was based on the Preferred Reporting Items for Systematic Reviews and Meta-analyses (PRISMA) statement [10] (Fig. 1).

### Inclusion and exclusion criteria

The inclusion criteria for studies were as follows: (1) case-control studies discussing the relationship between ACE I/D, AGT T704C, AT1R A1166C polymorphisms, and preeclampsia risk; (2) the diagnostic criteria for preeclampsia were defined as gestational hypertension, assessed as SBP > 140 mmHg, DBP > 90 mmHg, and/or rise in SBP > 30 mmHg or DBP > 15 mmHg on at least two occasions 6 h apart, following 20 weeks of gestation, with marked proteinuria (> 300 mg/24 h), or > 2+ proteinuria as tested by the dipstick method [5, 11, 12]; (3) the frequencies of the related polymorphisms in patients and controls could be retrieved to calculate odds ratio with 95% confidence intervals and to assess Hardy-Weinberg equilibrium. The exclusion criteria were (1) reviews or case reports or animal studies; (2) studies without reporting detailed genotype data; and (3) duplicated studies.

### Data extraction and quality assessment

The following information from eligible studies were extracted by the first two authors: the first author’ name, publication year, country, geography, ethnicity, PE maternal age, gestational weeks, PE degree, the genotype distributions and alleles in the patient and control groups, the result of the Hardy-Weinberg equilibrium, and the scores for quality assessment. For gestational weeks, early-onset PE was defined as gestational age (GA) between 20 and 33 weeks and 6 days, and late-onset PE was defined as GA 34 weeks and above. Severe PE was defined as severe hypertension (blood pressure ≥ 160/110 mmHg at least twice in a 24-h period) and/or severe proteinuria (5 g/24 h), or as hypertension with multiorgan involvement including fetal growth restriction or HELLP syndrome (hemolysis, elevated liver enzymes, and low platelet count) [13]. Any disagreement was resolved by group discussion with the corresponding author. The qualities of included studies were assessed by all the authors in accordance with the modified Newcastle-Ottawa Scale (NOS) (Table S1) [14]. Studies with scores of 7 points or higher were considered to be of high quality.

### Statistical analysis

The odds ratio (OR) and 95% confidence interval (95%CI) were calculated to investigate the effect strength of the associations between ACE I/D, AGT T704C, AT1R A1166C polymorphisms, and preeclampsia risk. The following genetic models were used: allelic genetic model (ACE I/D: D VS I; AGT T704C: C VS T; AT1R A1166C: C VS A), dominant genetic model (ACE I/D: DD + DI VS II; AGT T704C: CC + CT VS TT; AT1R A1166C: CC + CA VS AA), recessive genetic model (ACE I/D: DD VS DI + II; AGT T704C: CC VS CT + TT; AT1R A1166C: CC VS CA + AA), heterozygote genetic model (ACE I/D: DI VS II; AGT T704C: CT VS TT; AT1R A1166C: CA VS AA), and homozygote genetic model (ACE I/D: DD VS II; AGT T704C: CC VS TT; AT1R A1166C: CC VS AA). The Hardy-Weinberg equilibrium was assessed by the chi-squared test for every study in the control group. Heterogeneity in the meta-analysis was determined by the Cochrane’s Q-statistic test, and the inconsistency was quantified with the I^2^ statistic (I^2^ value more than 50% or *P* value less than 0.10 was considered significant heterogeneity and the random effect model was used, otherwise, the fixed-effect model was used). Sensitivity analysis was performed by omitting one study at a time to assess the influence of each study on the pooled results. Subgroup analysis was conducted, stratifying by geography (Asian, Europe, Africa, America and Australia), ethnicity (Caucasoid, Mongoloid, Black, Mixed race), gestational week (early-onset, late-onset, mixed), PE degree (severe, mild, not mentioned), and patient sample size (less than 100, between 100 and 200, more than 200). Publication bias was evaluated by a visual inspection of funnel plot and Egger’s test [15]. If publication bias existed, the “trim and fill” method was used; this method conservatively imputes hypothetical negative unpublished studies to mirror the positive studies that cause funnel plot asymmetry to further assess the possible effect of publication bias [16, 17]. All analyses were performed by Review Manager 5.3 and STATA 12.0 software packages and *P* < 0.5 was considered statistically significant.

#### Trial sequential analysis

TSA (trial sequential analysis) (The Copenhagen Trial Unit, Center for Clinical Intervention Research, Denmark) is a methodology that combines an information size calculation (accumulated sample sizes of all included trials) to reduce type I error and type II error for a meta-analysis with the threshold of statistical significance (http://www.ctu.dk/tsa). TSA was introduced into our meta-analysis. The required information size was calculated based on an overall type I error of 5%, a power of 90%, and a relative risk reduction (RRR) assumption of 10%.

## Results

### The characteristics of eligible studies

Table 1 and Fig. 1 show the main characteristics of the included studies and the study selection flow chart, respectively. A total of forty studies were finally included in our meta-analysis [1, 5, 7–9, 18–52], among which thirty-four studies involving 3977 patients and 7065 controls regarded the ACE I/D polymorphism, eighteen studies involving 1814 patients and 2892 controls regarded associations with AGT T704C polymorphism, and twelve studies involving 2391 cases and 6604 controls regarded the AT1R A1166C polymorphism.

**Table 1:** Characteristic of included studies regarding the associations between ACE insertion/deletion, AGT T704C, AT1R A1166C polymorphims and PE risk

										PE			CONTROL			Quality	
Author	Year	Country	Geography	Ethnicity	PE Maternal Age (years)	Gastation weeks	PE degree	PE	CONTROL	11	12	22	11	12	22	Scores	HWE
ACE insertion/deletion (I/D); 1 for I, 2 for D																	
Aung 1	2018	South Africa	South Africa	Neroid race	30.0 ± ?	Early-onset	Not mentioned	187	244	21	83	83	30	103	111	9	0.424
Aung 2	2018	South Africa	South Africa	Neroid race	26.0 ± ?	Late-onset	Not mentioned	170	244	12	79	79	30	103	111	9	0.424
Gonzalez-Garrido	2017	Mexico	South America	Mixed race	24.77 ± 5.20	Late-onset	Not mentioned	66	37	9	34	23	17	16	4	8	0.935
Ma	2015	China	East Asian	Mongoloid race	28.7 ± 3.6	Mixed	Not mentioned	188	273	90	84	14	122	115	36	9	0.285
Jahan	2014	India	South Asian	Caucasoid race	23.08 ± 3.73	Mixed	Not mentioned	206	206	36	61	109	29	113	64	9	0.063
Rahimi 1	2013	Iran	West Asian	Caucasoid race	29.3 ± 6.4	Mixed	Severe	70	100	11	16	43	16	42	42	8	0.322
Rahimi 2	2013	Iran	West Asian	Caucasoid race	29.0 ± 5.7	Mixed	Mild	128	100	14	33	81	16	42	42	9	0.322
Bereketoglu	2012	Turkey	West Asian	Caucasoid race	29.0 ± 7.04	Mixed	Not mentioned	120	114	17	51	52	16	68	30	7	0.024
Atalay	2012	Turkey	West Asian	Caucasoid race	29.11 ± 5.47	Mixed	Not mentioned	63	85	6	25	32	20	43	22	8	0.910
Salimi	2011	Iran	West Asian	Caucasoid race	27.2 ± 7.8	Mixed	Not mentioned	125	132	18	64	43	46	49	37	7	0.004
Xu	2012	China	East Asian	Mongoloid race	None*	Mixed	Not mentioned	50	50	9	24	17	20	20	10	8	0.239
Aggarwal 1	2011	India	South Asian	Caucasoid race	25.8 ± ?	Mixed	Severe	90	200	19	46	25	59	111	30	9	0.058
Aggarwal 2	2011	India	South Asian	Caucasoid race	26.1 ± ?	Mixed	Mild	110	200	37	48	25	59	111	30	9	0.058
Uma 1	2010	United Kingdom	West Europe	Caucasoid race	29.0 ± ?	Early-onset	Not mentioned	22	105	2	8	12	22	61	22	7	0.097
Uma 2	2010	United Kingdom	West Europe	Caucasoid race	29.0 ± ?	Late-onset	Not mentioned	38	105	12	19	7	22	61	22	7	0.097
Yue 1	2011	China	East Asian	Mongoloid race	None	Early-onset	Not mentioned	17	44	10	3	4	24	14	6	7	0.118
Yue 2	2011	China	East Asian	Mongoloid race	None	Late-onset	Not mentioned	26	44	13	5	8	24	14	6	7	0.118
Aggarwal 3	2010	India	South Asian	Caucasoid race	25.7 ± 3.8	Mixed	Not mentioned	120	118	38	66	16	45	54	19	8	0.679
Deng	2010	China	East Asian	Mongoloid race	None	Mixed	Not mentioned	50	100	14	16	20	23	57	20	8	0.158
Mando 1	2009	Italy	South Europe	Caucasoid race	33.4 ± 4.8	Mixed	Severe	119	410	15	50	54	72	187	151	9	0.287
Mando 2	2009	Italy	South Europe	Caucasoid race	33.4 ± 4.8	Mixed	Mild	78	410	6	46	26	72	187	151	9	0.287
Cui 1	2008	China	East Asian	Mongoloid race	None	Early-onset	Severe	36	40	9	19	8	17	19	4	7	0.694
Cui 2	2008	China	East Asian	Mongoloid race	None	Late-onset	Severe	27	40	10	14	3	17	19	4	7	0.694
Jiang	2008	China	East Asian	Mongoloid race	None	Mixed	Not mentioned	55	70	12	29	14	8	30	32	7	0.810
Miskovic	2008	Croatia	South Europe	Caucasoid race	31.4 ± 6.1	Mixed	Not mentioned	60	50	10	24	26	10	26	14	7	0.741
Zhan 1	2008	China	East Asian	Mongoloid race	None	Mixed	Severe	53	60	16	14	23	26	24	10	7	0.282
Zhan 2	2008	China	East Asian	Mongoloid race	None	Mixed	Mild	67	60	31	27	9	26	24	10	7	0.282
Benedetto	2007	Italy	South Europe	Caucasoid race	31.0 ± 4.0	Mixed	Not mentioned	120	103	24	50	46	13	54	35	8	0.264
Li	2007	China	East Asian	Mongoloid race	29.0 ± ?	Mixed	Not mentioned	133	105	50	46	37	49	31	25	7	0.000
Songa	2007	China	East Asian	Mongoloid race	None	Mixed	Not mentioned	45	45	7	21	17	9	23	13	7	0.839
Lia	2006	China	East Asian	Mongoloid race	None	Mixed	Not mentioned	82	45	24	33	25	11	19	15	7	0.318
Wang	2006	USA	North America	Mixed race	29.0 ± 7.2	Mixed	Not mentioned	123	1025	48	59	16	380	454	191	9	0.008
Kobashi	2005	Japan	East Asian	Mongoloid race	29.1 ± 0.5	Late-onset	Not mentioned	122	547	51	52	19	291	120	136	9	0.000
Kaur	2005	India	South Asian	Caucasoid race	24.9 ± 2.8	Late-onset	Not mentioned	12	50	3	2	7	9	26	15	7	0.696
Gurdol	2004	Turkey	West Asian	Caucasoid race	28.0 ± ?	Mixed	Not mentioned	95	89	17	31	47	21	37	31	8	0.136
Kim	2004	South Korea	East Asian	Mongoloid race	30.6 ± 5.7	Mixed	Not mentioned	188	210	66	72	50	62	98	50	9	0.357
Choi	2004	South Korea	East Asian	Mongoloid race	30.2 ± 4.5	Mixed	Not mentioned	100	100	26	38	36	34	52	14	8	0.405
Roberts 1	2004	South Africa	South Africa	Neroid race	26.3 ± ?	Early-onset	Not mentioned	67	338	8	29	30	44	142	152	7	0.238
Roberts 2	2004	South Africa	South Africa	Neroid race	26.3 ± ?	Late-onset	Not mentioned	204	338	23	86	95	44	142	152	9	0.238
Galao	2004	Brazil	South America	Mixed race	21.0 ± 4.1	Mixed	Not mentioned	51	71	12	23	16	17	33	21	8	0.570
Mello	2003	Italy	South Europe	Caucasoid race	29.0 ± ?	Mixed	Not mentioned	48	58	3	20	25	20	26	12	8	0.512
Bouba	2003	Greece	South Europe	Caucasoid race	31.0 ± ?	Mixed	Not mentioned	41	102	5	19	17	21	52	29	8	0.794
Heiskanen	2001	Finland	North Europe	Caucasoid race	None	Mixed	Not mentioned	133	115	31	59	43	26	58	31	9	0.909
Morgan	1999	United Kingdom	West Europe	Caucasoid race	28.8 ± 5.6	Mixed	Not mentioned	72	83	18	31	23	22	36	25	8	0.231
AGT T704C; 1 for T, 2 for C																	
Zitouni	2018	Tunisia	North Africa	Caucasoid race	30.6 ± 5.9	Mixed	Not mentioned	272	278	137	109	26	176	90	12	9	0.908
Shahvaisizadeh 1	2014	Iran	West Asian	Caucasoid race	29.6 ± 6.0	Mixed	Severe	74	100	19	37	18	31	41	28	8	0.073
Shahvaisizadeh 2	2014	Iran	West Asian	Caucasoid race	29.6 ± 6.0	Mixed	Mild	75	100	23	34	18	31	41	28	8	0.073
Groten 1	2014	Germany	Central Europe	Caucasoid race	None	Mixed	Severe	27	175	11	12	4	57	83	35	7	0.632
Groten 2	2014	Germany	Central Europe	Caucasoid race	None	Mixed	Mild	47	175	16	21	10	57	83	35	7	0.632
Groten 3	2014	Germany	Central Europe	Neroid race	None	Mixed	Severe	16	131	0	3	13	0	22	109	7	0.294
Groten 4	2014	Germany	Central Europe	Neroid race	None	Mixed	Mild	65	131	1	10	54	0	22	109	8	0.294
Radkov	2013	Russia	East Europe	Caucasoid race	26.5 ± 4.8	Mixed	Not mentioned	124	72	28	53	43	24	40	8	8	0.152
Coral-Vazquez	2013	Mexico	South America	Mixed race	25.1 ± 5.4	Mixed	Severe	230	352	11	72	147	20	122	210	9	0.682
Song	2013	China	East Asian	Mongoloid race	28.5 ± 2.2	Early-onset	Not mentioned	92	100	8	48	36	52	28	20	7	0.000
Aggarwal 1	2011	India	South Asian	Caucasoid race	25.8 ± ?	Mixed	Severe	90	200	18	51	21	35	116	49	8	0.019
Aggarwal 2	2011	India	South Asian	Caucasoid race	26.1 ± ?	Mixed	Mild	110	200	17	65	28	35	116	49	9	0.019
Aggarwal 4	2010	India	South Asian	Caucasoid race	25.7 ± 3.8	Mixed	Not mentioned	120	118	7	55	58	4	27	87	8	0.306
Jenkins 1	2008	USA	North America	Caucasoid race	28.1 ± 5.8	Mixed	Not mentioned	152	238	45	77	30	80	119	39	8	0.637
Jenkins 2	2008	USA	North America	Neroid race	21.3 ± 6.1	Mixed	Not mentioned	18	202	0	4	14	8	69	125	8	0.690
Songa	2007	China	East Asian	Mongoloid race	None	Mixed	Not mentioned	45	45	7	23	15	13	25	7	8	0.379
Procopciuc 1	2002	Romania	South Europe	Caucasoid race	29.20 ± 5.35	Mixed	Severe	5	6	2	2	1	3	2	1	7	0.540
Procopciuc 2	2002	Romania	South Europe	Caucasoid race	22.88 ± 1.36	Mixed	Mild	8	6	1	7	0	3	2	1	7	0.540
Bashford	2001	USA	North America	Caucasoid race	25.0 ± ?	Mixed	Not mentioned	68	50	5	28	35	1	28	21	6	0.018
Morgan	1999	United Kingdom	West Europe	Caucasoid race	None	Mixed	Not mentioned	43	84	12	21	10	22	43	19	7	0.818
Guo 1	1997	China	East Asian	Mongoloid race	None	Mixed	Not mentioned	75.6	48	4	23	49	3	18	27	7	0.999
Guo 2	1997	Australia	Australia	Caucasoid race	None	Mixed	Not mentioned	57.57	81	14	25	18	35	30	16	7	0.052
AT1R 1166A/C; 1 for A, 2 for C																	
Kvehaugen 1	2013	Norway	North Europe	Caucasoid race	26.6 ± ?	Early-onset	Not mentioned	71	2309	40	22	9	1139	975	195	8	0.501
Kvehaugen 2	2013	Norway	North Europe	Caucasoid race	26.6 ± ?	Late-onset	Not mentioned	1071	2309	548	433	90	1139	975	195	9	0.501
Rahimi 1	2013	Iran	West Asian	Caucasoid race	29.3 ± 6.4	Mixed	Severe	59	92	46	13	0	67	21	4	8	0.178
Rahimi 2	2013	Iran	West Asian	Caucasoid race	29.0 ± 5.7	Mixed	Mild	122	92	83	36	3	67	21	4	8	0.178
Salimi	2011	Iran	West Asian	Caucasoid race	27.2 ± 7.8	Mixed	Not mentioned	125	132	109	15	1	118	12	2	7	0.021
Deng	2010	China	East Asian	Mongoloid race	None	Mixed	Not mentioned	50	100	39	11	0	94	5	1	6	0.009
Akbar 1	2009	United Kingdom	West Europe	Mixed race	31.88 ± ?	Mixed	Not mentioned	67	119	63	4	0	98	18	3	7	0.070
Akbar 2	2009	Pakistan	South Asian	Caucasoid race	27.26 ± ?	Mixed	Not mentioned	121.878	188.811	99	21	2	156	32	1	8	0.638
Akbar 3	2009	United Kingdom	West Europe	Caucasoid race	31.85 ± ?	Mixed	Not mentioned	47	118	22	18	7	69	42	7	7	0.856
Benedetto	2007	Italy	South Europe	Caucasoid race	31.0 ± 4.0	Mixed	Not mentioned	120	103	64	46	10	53	40	10	8	0.547
Li	2007	China	East Asian	Mongoloid race	29.0 ± ?	Mixed	Not mentioned	133	105	109	23	1	94	10	1	8	0.234
Songa	2007	China	East Asian	Mongoloid race	None	Mixed	Not mentioned	45	45	26	11	8	25	15	5	7	0.256
Seremak-Mrozikiewicz	2005	Poland	East Europe	Caucasoid race	29.3 ± 5.6	Mixed	Not mentioned	47	113	23	21	3	64	46	3	7	0.113
Roberts 1	2004	South Africa	South Africa	Neroid race	26.3 ± ?	Early-onset	Not mentioned	67	338	67	0	0	338	0	0	6	0.000
Roberts 2	2004	South Africa	South Africa	Neroid race	26.3 ± ?	Late-onset	Not mentioned	204	338	204	0	0	338	0	0	7	0.000
Bouba	2003	Greece	South Europe	Caucasoid race	31.0 ± ?	Mixed	Not mentioned	41	102	25	11	5	58	37	7	8	0.741

*The PE maternal age is unavailable from the original article. *ACE*, angiotensin converting enzyme; *AGT*, angiotensinogen; *AT1R*, angiotensin II type 1 receptor; *PE*, preeclampsia; *HWE*, Hardy Weinberg equilibrium

### Meta-analysis results

Table 2 summarizes the overall and subgroup results regarding the associations between the ACE I/D, AGT T704C, and AT1R A1166C polymorphisms and PE risk. Extensive significant associations were observed for ACE I/D and AGT T704C polymorphisms; however, for the AT1R A1166C polymorphism, no association was detected.

**Table 2:** Overall and subgroup analysis of associations between ACE insertion/deletion, AGT T704C, AT1R A1166C polymorphisms, and PE risk

		Allelic genetic model					Dominant genetic model					Recessive genetic model					Heterozygote genetic model					Homozygote genetic model				
Subgroup	*N*	OR[95%CI]	P*	Effect model	I2	P#	OR[95%CI]	P*	Effect model	I2	P#	OR[95%CI]	P*	Effect model	I2	P#	OR[95%CI]	P*	Effect model	I2	P#	OR[95%CI]	P*	Effect model	I2	**P#**
ACE insertion/deletion (I/D)																										
Overall	39	*1.29 [1.16, 1.44]*	*0.000*	*R*	*60*	*0.000*	*1.17 [1.05, 1.31]*	*0.006*	*F*	*39*	*0.007*	*1.52 [1.18, 1.94]*	*0.001*	*R*	*82*	*0.000*	1.01 [0.90, 1.14]	0.820	F	35	0.020	*1.55 [1.26, 1.91]*	*0.000*	*R*	*51*	*0.000*
Geography																										
Asian	23	*1.31 [1.13, 1.53]*	*0.000*	*R*	*61*	*0.000*	1.10 [0.93, 1.31]	0.250	R	23	0.160	*1.80 [1.33, 2.43]*	*0.000*	*R*	*74*	*0.000*	0.90 [0.74, 1.08]	0.240	R	23	0.160	*1.53 [1.16, 2.01]*	*0.002*	*R*	*52*	*0.002*
Europe	10	*1.33 [1.05, 1.67]*	*0.020*	*R*	*63*	*0.003*	1.33 [0.88, 2.01]	0.170	R	57	0.010	1.20 [0.68, 2.12]	0.520	R	86	0.000	1.15 [0.78, 1.69]	0.480	R	46	0.050	*1.68 [1.06, 2.66]*	*0.030*	*R*	*57*	*0.010*
Africa	4	1.07 [0.92, 1.24]	0.410	R	0	0.910	1.26 [0.91, 1.73]	0.160	R	0	0.690	0.97 [0.59, 1.58]	0.900	R	83	0.000	1.28 [0.91, 1.79]	0.150	R	0	0.680	1.24 [0.89, 1.73]	0.210	R	0	0.740
America	2	1.81 [0.60, 5.42]	0.290	R	87	0.005	2.31 [0.45, 11.76]	0.310	R	85	0.010	3.00 [0.39, 23.26]	0.290	R	89	0.003	1.96 [0.50, 7.74]	0.340	R	76	0.040	3.27 [0.34, 31.44]	0.300	R	87	0.006
Ethnicity																										
Caucasoid race	19	*1.39 [1.21, 1.60]*	*0.000*	*R*	*52*	*0.004*	1.24 [0.98, 1.56]	0.070	R	42	0.030	1.24 [0.98, 1.56]	0.020	R	86	0.000	0.99 [0.77, 1.29]	0.960	R	48	0.010	*1.68 [1.30, 2.17]*	*0.000*	*R*	*39*	*0.040*
Mongoloid race	14	1.20 [0.96, 1.51]	0.110	R	66	0.000	1.06 [0.84, 1.33]	0.630	R	26	0.170	1.06 [0.84, 1.33]	0.030	R	67	0.000	0.91 [0.74, 1.11]	0.330	R	0	0.500	1.40 [0.90, 2.16]	0.130	R	63	0.000
Black race	4	1.07 [0.92, 1.24]	0.410	R	0	0.910	1.26 [0.91, 1.73]	0.160	R	0	0.690	1.26 [0.91, 1.73]	0.900	R	83	0.000	1.28 [0.91, 1.79]	0.150	R	0	0.680	1.24 [0.89, 1.73]	0.210	R	0	0.740
Mixed race	2	1.81 [0.60, 5.42]	0.290	R	87	0.005	2.31 [0.45, 11.76]	0.310	R	85	0.010	2.31 [0.45, 11.76]	0.290	R	89	0.003	1.96 [0.50, 7.74]	0.340	R	76	0.040	3.27 [0.34, 31.44]	0.300	R	87	0.006
Gestation weeks																										
Early-onset	5	1.30 [0.92, 1.83]	0.130	R	54	0.070	1.26 [0.86, 1.86]	0.240	R	0	0.560	0.98 [0.47, 2.05]	0.960	R	77	0.002	1.18 [0.78, 1.79]	0.440	R	0	0.710	1.61 [0.89, 2.92]	0.110	R	34	0.190
Late-onset	7	1.29 [0.96, 1.74]	0.090	R	59	0.020	1.35 [0.82, 2.23]	0.240	R	57	0.030	1.35 [0.81, 2.25]	0.260	R	67	0.006	1.16 [0.68, 1.99]	0.580	R	56	0.030	1.69 [0.94, 3.03]	0.080	R	53	0.050
Mixed	27	*1.29 [1.13, 1.48]*	*0.000*	*R*	*64*	*0.000*	1.16 [0.98, 1.39]	0.090	R	41	0.020	*1.66 [1.22, 2.26]*	*0.001*	*R*	*84*	*0.000*	0.97 [0.81, 1.16]	0.740	R	35	0.040	*1.52 [1.19, 1.94]*	*0.000*	*R*	*56*	*0.000*
PE degree																										
Severe	6	*1.53 [1.28, 1.83]*	*0.000*	*R*	*0*	*0.590*	*1.50 [1.11, 2.04]*	*0.009*	*R*	*0*	*0.880*	1.59 [0.82, 3.11]	0.170	R	78	0.000	1.16 [0.83, 1.61]	0.380	R	0	0.610	*2.14 [1.49, 3.09]*	*0.000*	*R*	*0*	*0.610*
Mild	4	1.21 [0.90, 1.61]	0.210	R	55	0.090	1.21 [0.74, 1.98]	0.450	R	50	0.110	1.11 [0.27, 4.63]	0.880	R	95	0.000	1.08 [0.60, 1.96]	0.800	R	61	0.050	1.51 [0.99, 2.31]	0.060	R	4	0.370
Not mentioned	29	*1.26 [1.10, 1.45]*	*0.000*	*R*	*65*	*0.000*	1.16 [0.96, 1.41]	0.120	R	45	0.005	*1.57 [1.22, 2.02]*	*0.000*	*R*	*77*	*0.000*	1.00 [0.82, 1.21]	0.990	R	40	0.010	*1.47 [1.14, 1.90]*	*0.003*	*R*	*57*	*0.000*
Case sample size																										
< 100	26	*1.41 [1.19, 1.66]*	*0.000*	*R*	*61*	*0.000*	*1.37 [1.09, 1.73]*	*0.008*	*R*	*41*	*0.020*	*1.50 [1.05, 2.15]*	*0.020*	*R*	*80*	*0.000*	1.13 [0.89, 1.42]	0.310	R	31	0.070	*1.85 [1.37, 2.51]*	*0.000*	*R*	*52*	*0.001*
≥ 100 and < 200	11	1.13 [0.98, 1.31]	0.090	R	53	0.020	1.05 [0.87, 1.27]	0.610	R	23	0.220	1.46 [0.99, 2.17]	0.060	R	85	0.000	0.96 [0.79, 1.17]	0.700	R	19	0.260	1.24 [0.93, 1.66]	0.140	R	48	0.040
≥200	2	1.26 [0.92, 1.73]	0.150	R	63	0.100	0.95 [0.63, 1.44]	0.820	R	16	0.280	2.05 [0.68, 6.19]	0.200	R	94	0.000	0.71 [0.27, 1.86]	0.490	R	82	0.020	1.28 [0.85, 1.92]	0.230	R	0	0.740
AGT 704T/C																										
Overall	18	1.16 [0.96, 1.41]	0.120	R	60	0.000	*1.33 [1.12, 1.59]*	*0.001*	*F*	*0*	*0.510*	1.29 [0.86, 1.94]	0.210	R	80	0.000	*1.26 [1.05, 1.52]*	*0.010*	*F*	*0*	*0.840*	*1.44 [1.14, 1.83]*	*0.003*	*F*	*36*	*0.070*
Geography																										
Asian	5	0.98 [0.62, 1.57]	0.950	R	78	0.001	1.18 [0.80, 1.74]	0.410	R	0	0.560	1.68 [0.84, 3.33]	0.140	R	81	0.000	1.30 [0.85, 1.97]	0.220	R	0	0.950	1.08 [0.57, 2.05]	0.810	R	42	0.140
Europe	8	1.12 [0.84, 1.49]	0.430	R	28	0.200	1.08 [0.73, 1.60]	0.690	R	10	0.360	0.89 [0.30, 2.64]	0.830	R	87	0.000	0.97 [0.66, 1.41]	0.860	R	0	0.570	1.20 [0.57, 2.52]	0.630	R	45	0.090
Africa	1	1.63 [1.24, 2.15]	0.000	R	NA	NA	1.70 [1.21, 2.39]	0.002	R	NA	NA	2.40 [1.18, 4.85]	0.020	R	NA	NA	1.56 [1.09, 2.22]	0.020	R	NA	NA	2.78 [1.36, 5.72]	0.005	R	NA	NA
America	3	1.19 [0.97, 1.45]	0.090	R	0	0.550	1.21 [0.83, 1.76]	0.320	R	0	0.980	1.15 [0.57, 2.30]	0.700	R	76	0.020	1.13 [0.76, 1.68]	0.550	R	0	0.990	1.34 [0.84, 2.14]	0.210	R	0	0.960
Australia	1	1.89 [1.16, 3.06]	0.010	R	NA	NA	2.18 [1.05, 4.54]	0.040	R	NA	NA	1.63 [0.74, 3.58]	0.220	R	NA	NA	2.08 [0.92, 4.71]	0.080	R	NA	NA	2.81 [1.13, 7.02]	0.030	R	NA	NA
Ethnicity																										
Caucasoid race	12	1.11 [0.86, 1.44]	0.420	R	71	0.000	*1.30 [1.05, 1.60]*	*0.020*	*R*	*13*	*0.320*	1.03 [0.61, 1.72]	0.920	R	79	0.000	*1.28 [1.05, 1.56]*	*0.020*	*R*	*0*	*0.680*	1.35 [0.91, 2.01]	0.140	R	47	0.030
Mongoloid race	2	*1.60 [1.04, 2.44]*	*0.030*	*R*	*0*	*0.440*	1.83 [0.77, 4.31]	0.170	R	0	0.520	*4.43 [2.57, 7.62]*	*0.000*	*R*	*0*	*0.460*	1.43 [0.58, 3.51]	0.430	R	0	0.560	2.57 [0.91, 7.22]	0.070	R	7	0.300
Black race	3	1.13 [0.65, 1.95]	0.670	R	0	0.390	0.57 [0.06, 5.38]	0.630	R	7	0.300	1.07 [0.31, 3.76]	0.910	R	75	0.020	0.45 [0.05, 4.14]	0.480	R	0	0.390	0.63 [0.06, 7.09]	0.710	R	20	0.260
Mixed race	1	1.16 [0.87, 1.55]	0.300	R	NA	NA	1.20 [0.56, 2.55]	0.640	R	NA	NA	1.94 [1.40, 2.69]	0.000	R	NA	NA	1.07 [0.49, 2.37]	0.860	R	NA	NA	1.27 [0.59, 2.74]	0.540	R	NA	NA
PE degree																										
Severe	5	1.06 [0.85, 1.31]	0.600	R	0	0.770	1.09 [0.71, 1.66]	0.700	R	0	0.690	0.72 [0.27, 1.91]	0.500	R	82	0.000	1.12 [0.71, 1.75]	0.630	R	0	0.710	1.04 [0.63, 1.73]	0.870	R	0	0.770
Mild	4	0.97 [0.73, 1.28]	0.810	R	0	0.930	1.01 [0.60, 1.69]	0.980	R	10	0.340	0.90 [0.31, 2.62]	0.840	R	80	0.002	1.07 [0.54, 2.11]	0.860	R	28	0.240	0.88 [0.49, 1.56]	0.660	R	0	0.770
Not mentioned	9	1.32 [0.96, 1.82]	0.090	R	77	0.000	*1.49 [1.20, 1.85]*	*0.000*	*R*	*0*	*0.540*	*1.87 [1.08, 3.24]*	*0.030*	*R*	*81*	*0.000*	*1.36 [1.08, 1.70]*	*0.009*	*R*	*0*	*0.890*	*1.87 [1.21, 2.88]*	*0.005*	*R*	*4*	*0.070*
Case sample size																										
< 100	13	1.15 [0.96, 1.36]	0.120	R	6	0.380	1.19 [0.91, 1.56]	0.210	R	0	0.530	0.99 [0.55, 1.78]	0.970	R	79	0.000	1.19 [0.89, 1.60]	0.250	R	0	0.660	1.21 [0.86, 1.70]	0.280	R	0	0.470
≥ 100 and < 200	3	1.00 [0.47, 2.16]	0.990	R	92	0.000	1.25 [0.82, 1.90]	0.290	R	19	0.290	2.06 [0.71, 6.00]	0.180	R	90	0.000	1.15 [0.79, 1.66]	0.470	R	0	1.000	1.43 [0.44, 4.64]	0.550	R	80	0.006
≥ 200	2	1.38 [0.99, 1.92]	0.060	R	64	0.100	*1.60 [1.18, 2.19]*	*0.003*	*R*	*0*	*0.410*	*2.01 [1.50, 2.71]*	*0.000*	*R*	*0*	*0.590*	*1.46 [1.05, 2.02]*	*0.020*	*R*	*0*	*0.400*	1.90 [0.88, 4.10]	0.100	R	53	0.140
AT1R 1166 A/C																										
Overall	12	0.98 [0.90, 1.08]	0.730	F	28	0.170	0.95 [0.85, 1.07]	0.430	F	16	0.290	0.60 [0.29, 1.21]	0.150	R	78	0.000	0.94 [0.83, 1.06]	0.300	F	13	0.320	1.04 [0.83, 1.30]	0.740	F	0	0.480
Geography																										
Asian	5	1.11 [0.84, 1.45]	0.470	R	0	0.470	1.14 [0.84, 1.55]	0.400	R	0	0.540	1.15 [0.52, 2.57]	0.730	R	0	0.440	1.16 [0.84, 1.61]	0.360	R	0	0.460	1.02 [0.45, 2.32]	0.960	R	0	0.510
Europe	7	1.01 [0.82, 1.24]	0.940	R	45	0.090	0.93 [0.74, 1.16]	0.520	R	29	0.200	0.48 [0.19, 1.19]	0.110	R	85	0.000	0.89 [0.73, 1.08]	0.240	R	14	0.320	1.17 [0.82, 1.66]	0.380	R	19	0.290
Ethnicity																										
Caucasoid race	9	0.99 [0.90, 1.08]	0.780	R	0	0.500	0.95 [0.85, 1.08]	0.450	R	0	0.660	0.54 [0.24, 1.20]	0.130	R	82	0.000	0.94 [0.83, 1.07]	0.320	R	0	0.670	1.14 [0.82, 1.57]	0.440	R	13	0.330
Mongoloid race	2	1.35 [0.84, 2.18]	0.220	R	0	0.380	1.34 [0.66, 2.71]	0.420	R	36	0.210	1.76 [0.58, 5.28]	0.320	R	0	0.670	1.22 [0.45, 3.37]	0.690	R	63	0.100	1.40 [0.45, 4.35]	0.560	R	0	0.710
Mixed race	1	*0.27 [0.09, 0.81]*	*0.020*	*R*	*NA*	*NA*	*0.30 [0.10, 0.90]*	*0.030*	*R*	NA	NA	0.14 [0.01, 2.82]	0.200	R	NA	NA	0.35 [0.11, 1.07]	0.070	R	NA	NA	0.22 [0.01, 4.36]	0.320	R	NA	NA
Gestation weeks																										
Early-onset	1	0.93 [0.64, 1.35]	0.720	R	NA	NA	0.75 [0.47, 1.21]	0.250	R	NA	NA	*0.05 [0.02, 0.11]*	*0.000*	*R*	*NA*	*NA*	0.64 [0.38, 1.09]	0.100	R	NA	NA	1.31 [0.63, 2.75]	0.470	R	NA	NA
Late-onset	1	0.96 [0.85, 1.07]	0.430	R	NA	NA	0.93 [0.80, 1.07]	0.320	R	NA	NA	*0.50 [0.39, 0.66]*	*0.000*	*R*	*NA*	*NA*	0.92 [0.79, 1.07]	0.300	R	NA	NA	0.96 [0.73, 1.26]	0.760	R	NA	NA
Mixed	10	1.08 [0.86, 1.36]	0.490	R	34	0.140	1.07 [0.84, 1.36]	0.590	R	18.000	0.28	1.03 [0.65, 1.63]	0.910	R	0	0.770	1.05 [0.83, 1.33]	0.700	R	6	0.380	1.31 [0.81, 2.11]	0.270	R	1	0.430
PE degree																										
Severe	1	0.66 [0.33, 1.33]	0.250	R	NA	NA	0.76 [0.35, 1.63]	0.480	R	NA	NA	0.11 [0.01, 2.11]	0.140	R	NA	NA	0.90 [0.41, 1.98]	0.800	R	NA	NA	0.16 [0.01, 3.07]	0.220	R	NA	NA
Mild	1	1.11 [0.66, 1.86]	0.690	R	NA	NA	1.26 [0.69, 2.29]	0.450	R	NA	NA	0.77 [0.17, 3.51]	0.730	R	NA	NA	1.38 [0.74, 2.59]	0.310	R	NA	NA	0.61 [0.13, 2.80]	0.520	R	NA	NA
Not mentioned	10	1.05 [0.88, 1.25]	0.570	R	34	0.130	0.98 [0.81, 1.19]	0.840	R	25	0.220	0.63 [0.29, 1.39]	0.250	R	81	0.000	0.93 [0.77, 1.13]	0.460	R	19	0.270	1.08 [0.86, 1.36]	0.490	R	0	0.490
Case sample size																										
< 100	7	1.00 [0.73, 1.37]	1.000	R	49	0.070	0.90 [0.65, 1.25]	0.530	R	31	0.190	0.44 [0.11, 1.76]	0.250	R	85	0.000	0.82 [0.61, 1.11]	0.200	R	10	0.350	1.55 [0.95, 2.52]	0.080	R	3	0.400
≥ 100 and < 200	4	1.10 [0.85, 1.43]	0.460	R	0	0.530	1.17 [0.87, 1.58]	0.310	R	0	0.510	1.06 [0.52, 2.18]	0.870	R	0	0.930	1.20 [0.88, 1.65]	0.250	R	0	0.450	0.87 [0.42, 1.83]	0.720	R	0	0.720
≥ 200	1	0.96 [0.85, 1.07]	0.430	R	NA	NA	0.93 [0.80, 1.07]	0.320	R	NA	NA	*0.50 [0.39, 0.66]*	*0.000*	*R*	*NA*	*NA*	0.92 [0.79, 1.07]	0.300	R	NA	NA	0.96 [0.73, 1.26]	0.760	R	NA	NA

*P value for meta-analysis, # *P* value for heterogeneity test; F means the fixed effect model, R means the random effect model, *NA*, not available for the only one included study; *OR*, odds ratio; *CI*, confidence interval; *ACE*, angiotensin converting enzyme; *AGT*, angiotensinogen; *AT1R*, angiotensin II type 1 receptor; *PE*, preeclampsia

Significant results are in italics

#### AGT T704C polymorphism

As summarized in Table 2, the overall analysis indicated that the AGT T704C polymorphism was associated with PE risk in three genetic models (dominant genetic model: CC+CT VS TT: OR = 1.33, 95%CI = 1.12–1.59 (Fig. 3); heterozygote genetic model: OR = 1.26, 95%CI = 1.05–1.52: homozygote genetic model: OR = 1.44, 95%CI = 1.14–1.83). No heterogeneity was observed in the three genetic models. For subgroup analysis by geography, no significant association was detected (Fig. 4b). As stratified by ethnicity, the AGT T704C polymorphism was associated with PE risk both in Caucasoid and Mongoloid populations (Caucasoid: dominant genetic model: CC+CT VS TT: OR = 1.30, 95%CI = 1.05–1.60 (Fig. 5b); heterozygote genetic model: CT VS TT: OR = 1.28, 95%CI = 1.05–1.56. Mongoloid: allelic genetic model: C VS T: OR = 1.60, 95%CI = 1.04–.44; recessive genetic model: CCVS CT+TT: OR = 4.43, 95%CI = 2.57–7.62). No associations were also observed in the severe or the mild subgroup either. In the subgroup analysis by patient sample size, significant associations were detected in the dominant (CC+CT VS TT: OR = 1.60, 95%CI = 1.18–2.19), recessive (CC VS CT+TT: OR = 2.01, 95%CI = 1.50–2.71), and heterozygote (CT VS TT: OR = 1.46, 95%CI = 1.05–2.02) genetic model in more than 200 subgroups.

#### ACE I/D polymorphism

**Fig. 2:**
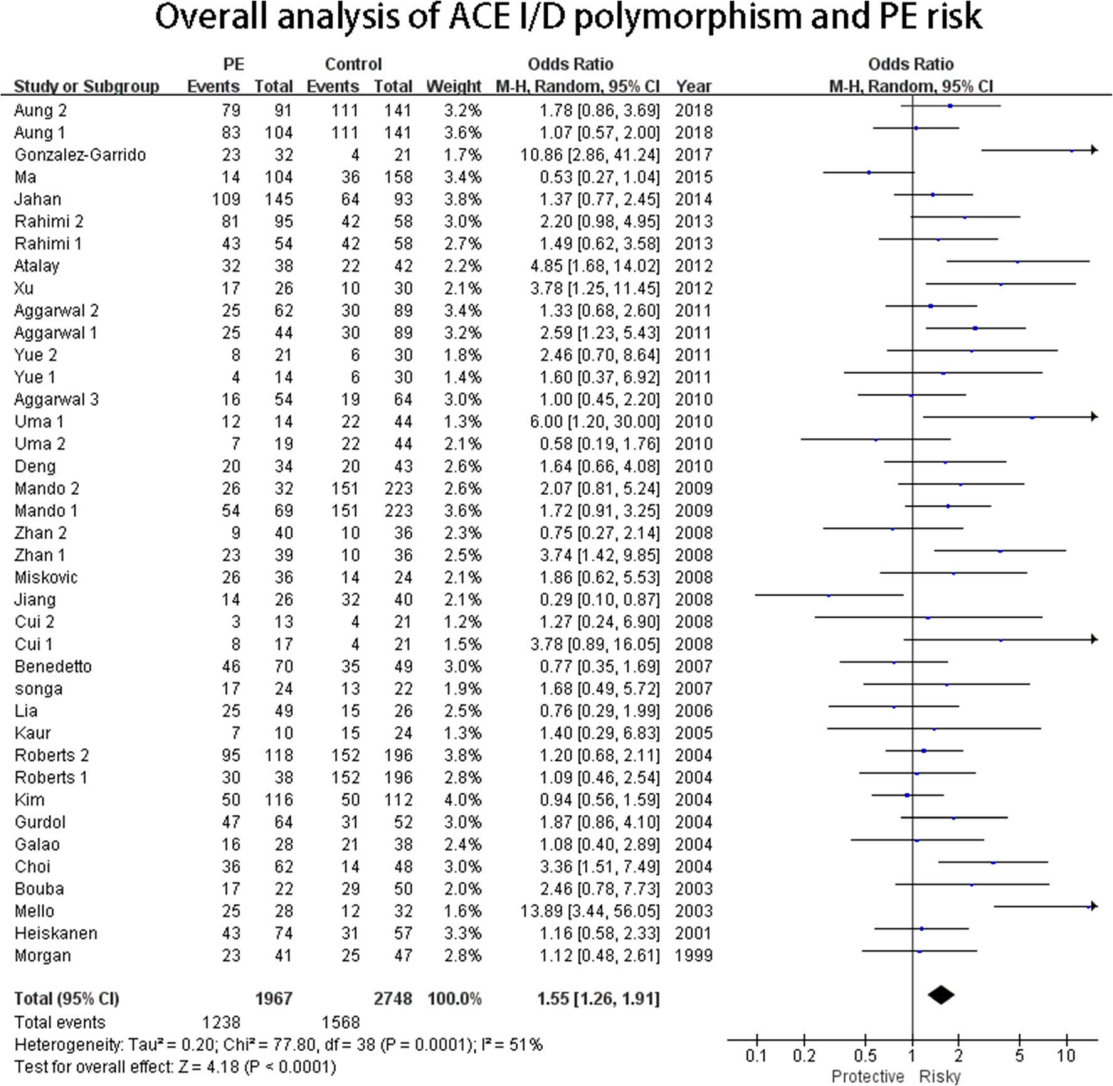
Overall analysis of ACE I/D polymorphism and PE risk

In the overall analysis, significant associations with significant heterogeneity were observed in the allelic genetic model (D VS I: OR = 1.29, 95%CI = 1.16–1.44), the dominant genetic model (DD+DI VS II: OR = 1.17, 95%CI = 1.05–1.31), the recessive genetic model (DD VS DI+II: OR = 1.52, 95%CI = 1.18–1.94), and the homozygote genetic model (DD VS II: OR = 1.55, 95%CI = 1.26–1.91) (Fig. 2). Galbraith plot analyses were performed to further explore the sources of heterogeneity, and the figure showed that the studies performed by Mello et al. [46], Gonzalez et al. [1], Choi et al. [45], Atalay et al. [26], Zhan1 et al. [32], Jiang et al. [34], and Ma et al. [18] primarily contributed to the heterogeneity. After excluding these studies, the heterogeneity decreased significantly (I2 = 21% and PHeterogeneity = 0.14 for D VS I; I2 = 6% and PHeterogeneity = 0.37 for DD+DI VS II; I2 = 14% and PHeterogeneity = 0.25 for DD VS DI+II; I2 = 0 and PHeterogeneity = 0.58 for DD VS II). For subgroup analysis stratified by geography, the ACE ID polymorphism was similarly associated with PE risk in three genetic models in the Asian population (allelic genetic model: D VS I: OR = 1.31, 95%CI = 1.13–1.53; recessive genetic model: DD VS DI+II: OR = 1.80, 95%CI = 1.33–2.43; homozygote genetic model: DD VS II: OR = 1.53, 95%CI = 1.16–2.01 (Fig. 4a)). Regarding the ethnicity subgroup analysis, significant associations were only observed in allelic (D VS I: OR = 1.39, 95%CI = 1.21–1.60) and homozygote genetic models (DD VS II: OR = 1.68, 95%CI = 1.30–2.17) in Caucasoid. However, for the subgroup analysis of gestational weeks, no significant association was detected in both early-onset and late-onset subgroups. In the severe PE subgroup, the ACE I/D polymorphism was associated with PE in allelic genetic (D VS I: OR = 1.53, 95%CI = 1.28–1.83), dominant (DD+DI VS II: OR = 1.50, 95%CI = 1.11–2.04), and homozygote (DD VS II: OR = 2.14, 95%CI = 1.49–3.09) genetic models. For the subgroup of patient sample size less than 100, wide associations with PE risk were observed in allelic (D VS I: OR = 1.41, 95%CI = 1.19–1.66), dominant (DD+DI VS II: OR = 1.37, 95%CI = 1.09–1.73), recessive (DD VS DI+II: OR = 1.50, 95%CI = 1.05–2.15), and homozygote (DD VS II: OR = 1.85, 95%CI = 1.37–2.51 (Fig. 5a)) genetic models..

**Fig. 3:**
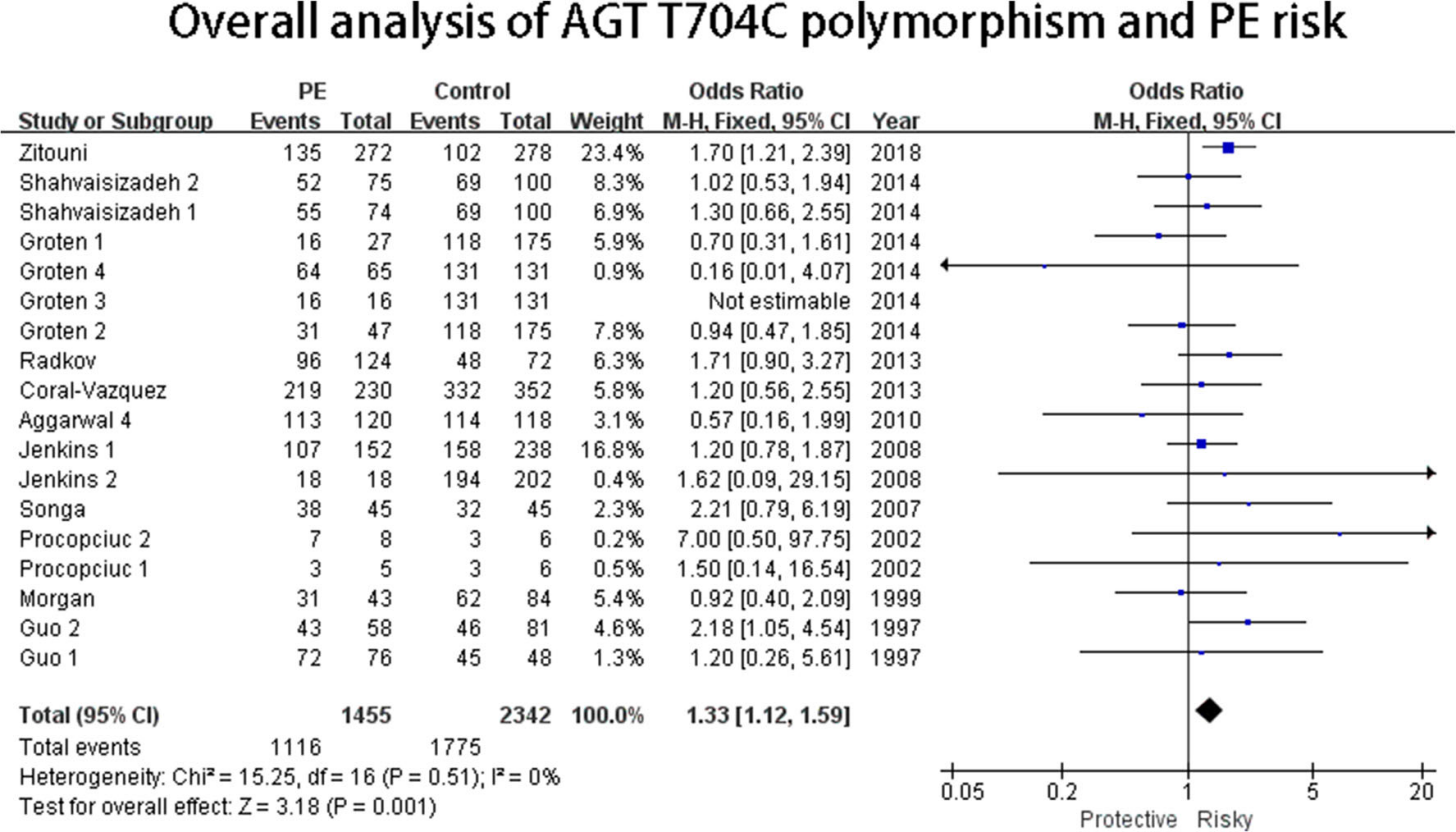
Overall analysis of AGT T704C polymorphism and PE risk

#### AT1R A1166C polymorphism

**Fig. 4:**
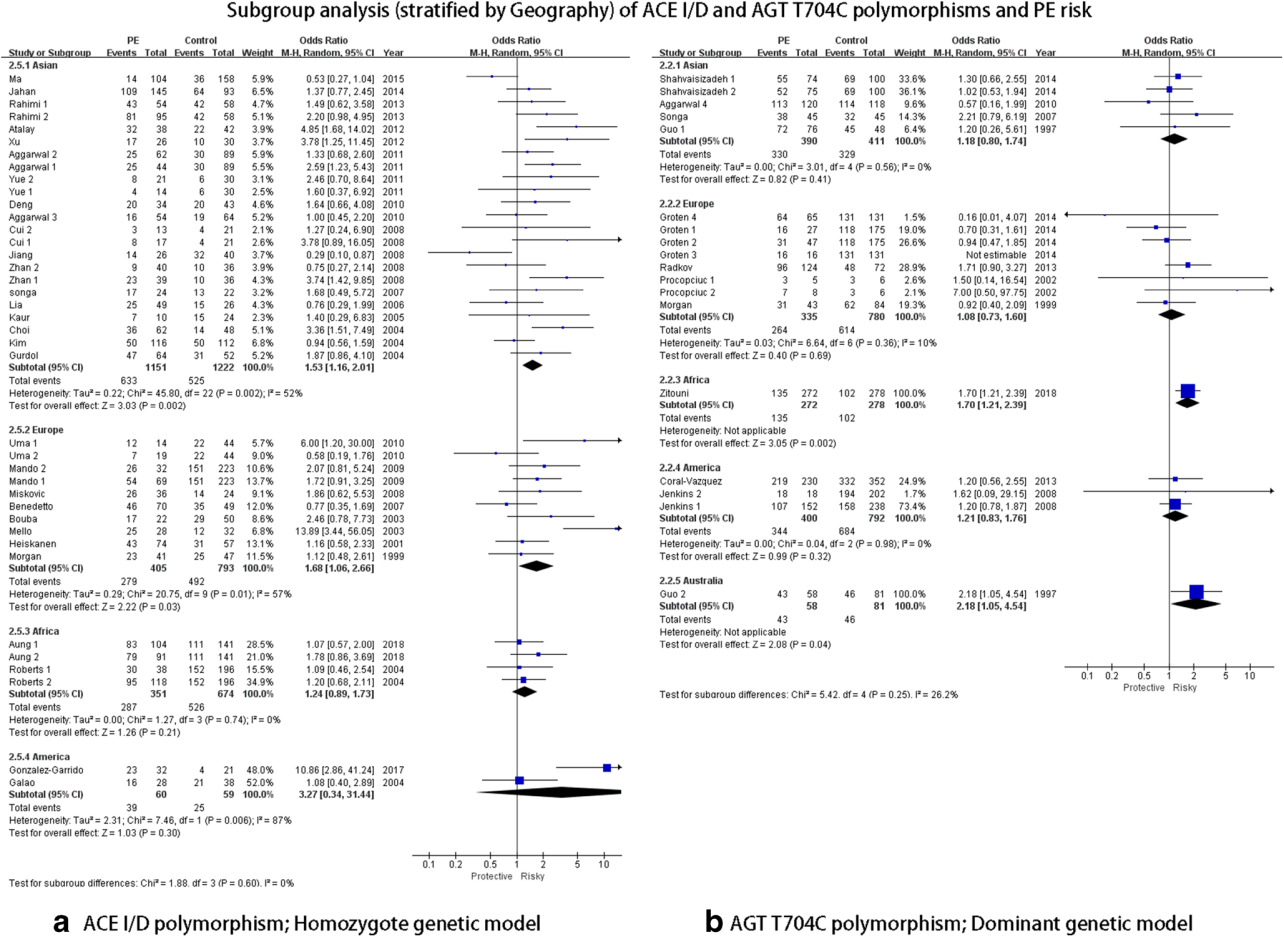
Subgroup analysis (stratified by geography) of ACE I/D and AGT T704C polymorphisms and PE risk

**Fig. 5:**
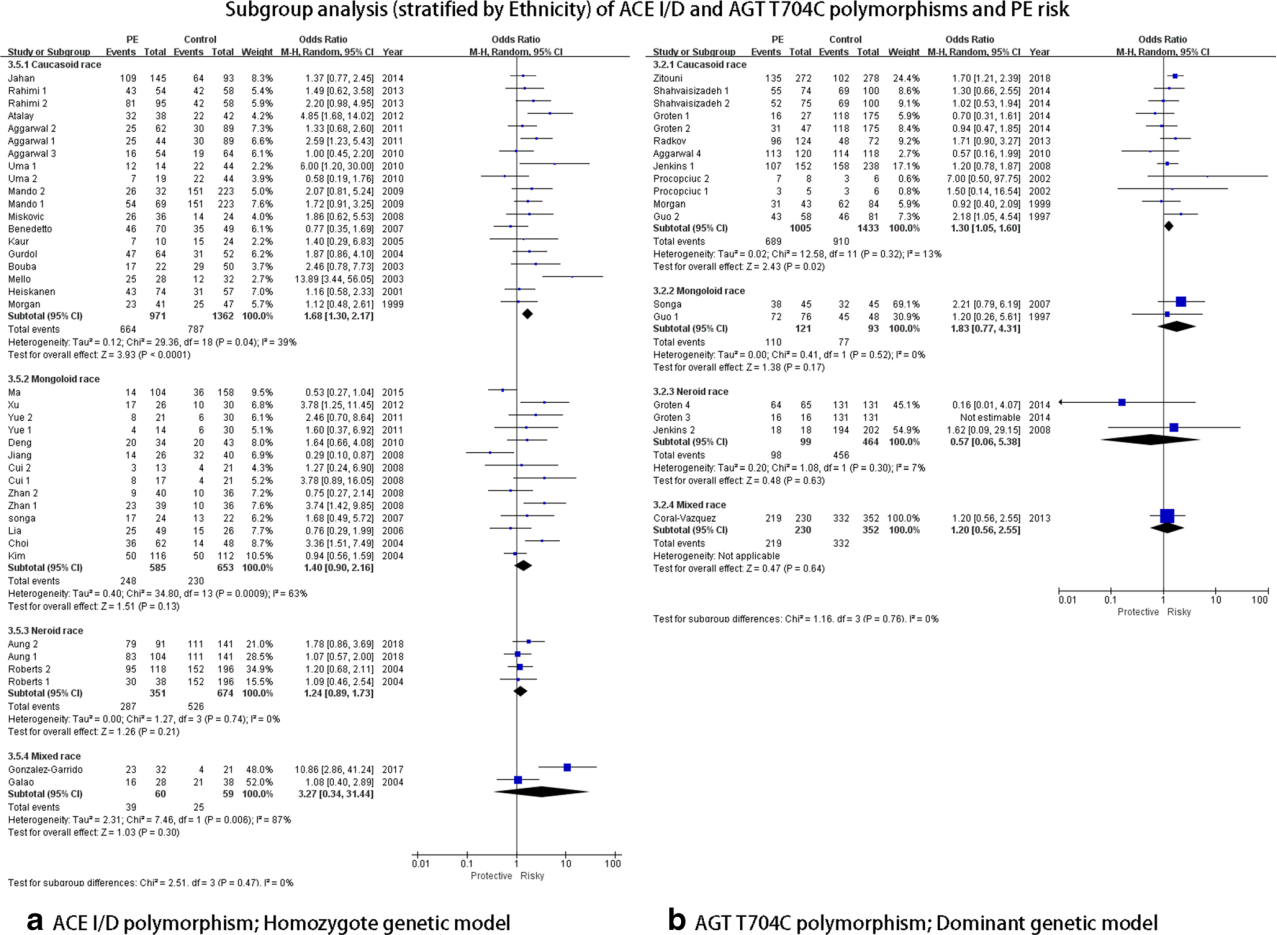
Subgroup analysis (stratified by ethnicity) of ACE I/D and AGT T704C polymorphisms and PE risk

As shown in Table 2, significant associations were observed in mixed race, early-onset, late-onset, and more than 200 subgroups; however, only one study was analyzed in these subgroups and the results required interpretation with caution (Figs. 4 and 5).

### Sensitivity analysis and publication bias

**Fig. 6:**
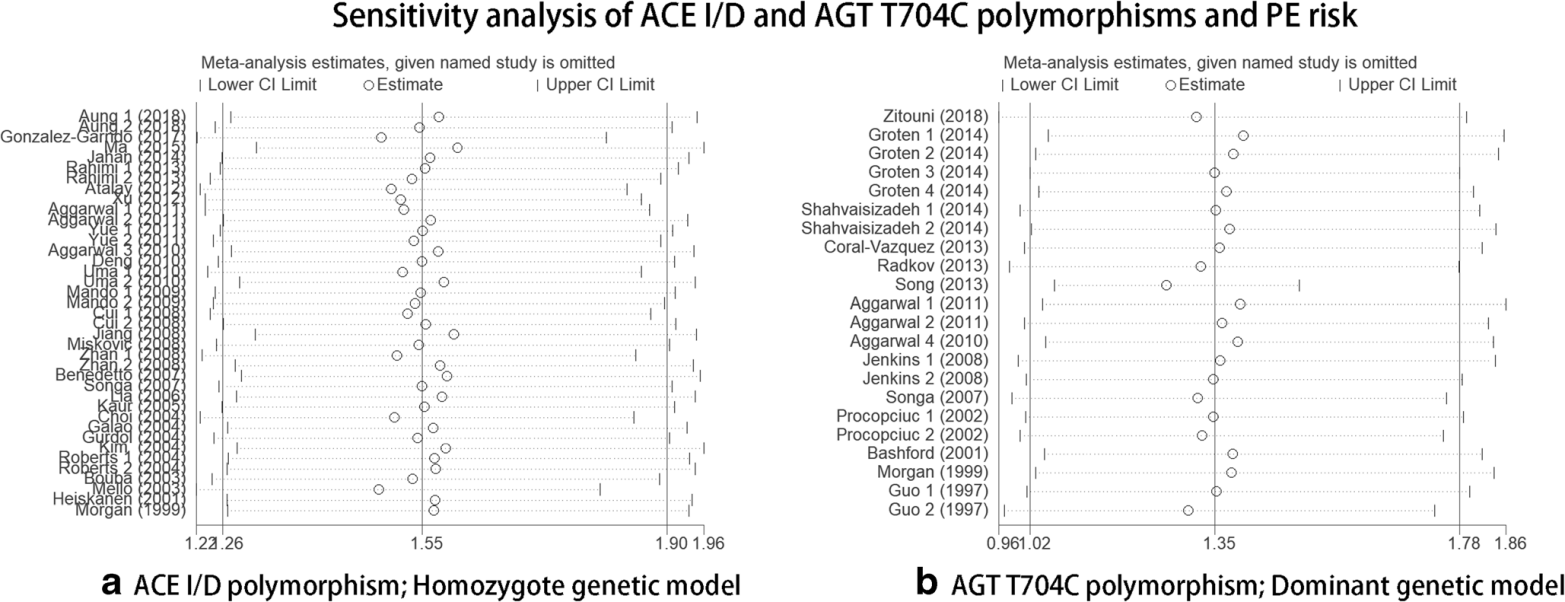
Sensitivity analysis of ACE I/D and AGT T704C polymorphisms and PE risk

**Fig. 7:**
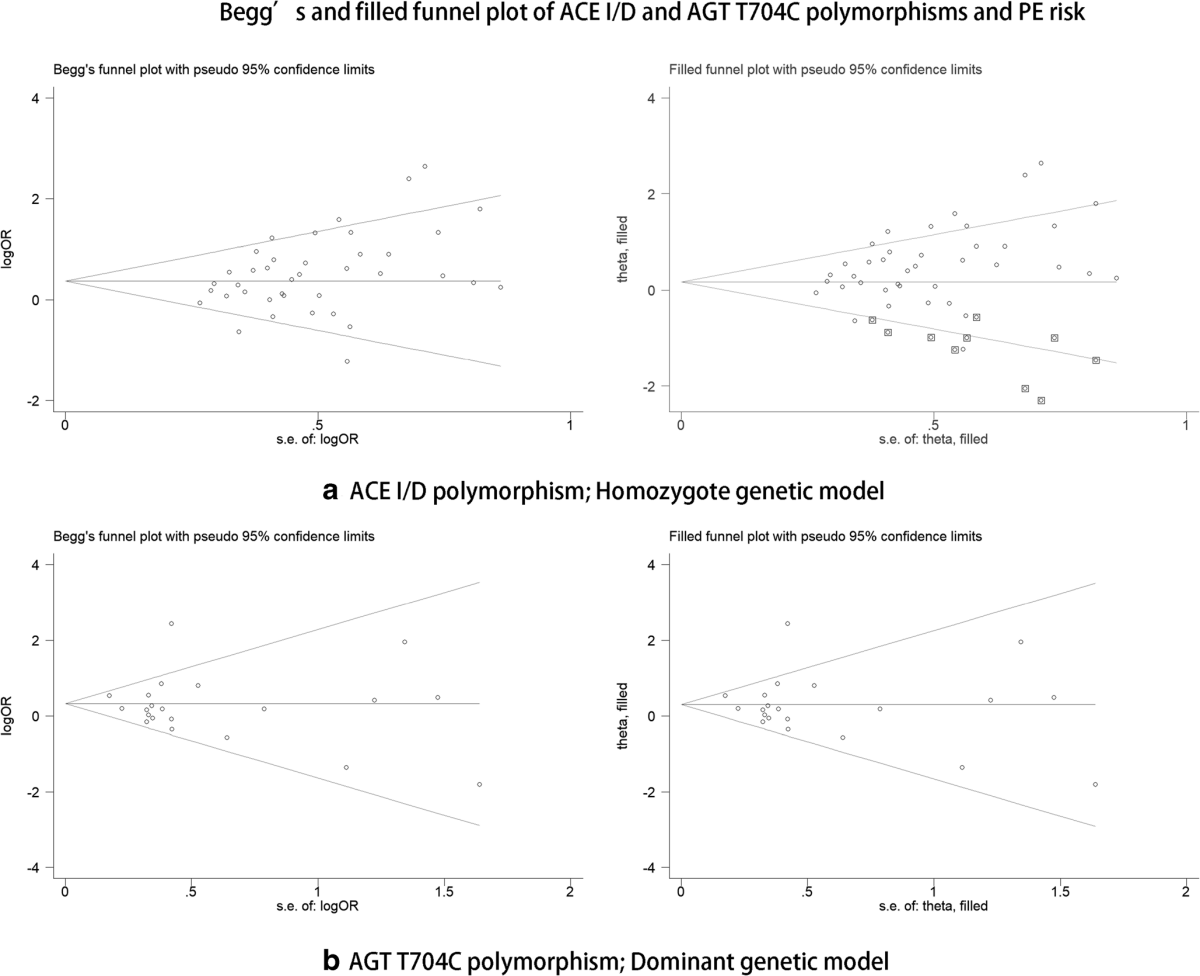
Begg’s and filled funnel plot of ACE I/D and AGT T704C polymorphisms and PE risk

Sensitivity analysis was performed, and every study was omitted one a time, without any effect on our overall statistical results, indicating that the results were stable and reliable (Fig. 6). Begg’s and Egger’s test were conducted to analyze publication bias (*P* = 0.015 for ACE I/D polymorphism; *P* = 0.627 for AGT T704C polymorphism) (Fig. 7). Our results indicated that publication bias was existed in ACE I/D polymorphism; therefore, we applied a sensitivity analysis using the trim and fill method [16], which conservatively imputed hypothetical negative unpublished studies to mirror the positive studies that cause funnel plot asymmetry; the imputed studies of ACE I/D polymorphism produced a symmetrical funnel plot [53] (Fig. 7a). The shape of funnel plot was symmetrical for the AGT T704C polymorphism (Fig. 7b), implying that there was no publication bias for this polymorphism.

### Trial sequential analysis

**Fig. 8:**
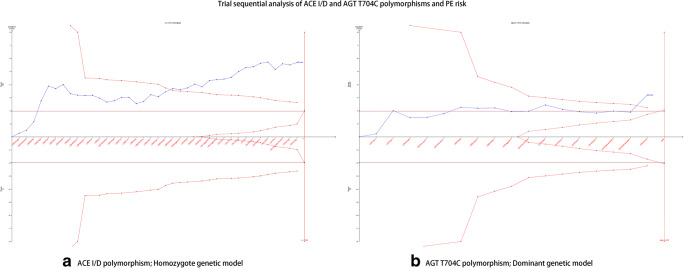
Trial sequential analysis of ACE I/D and AGT T704C polymorphisms and PE risk

We performed a TSA for the homozygote genetic model of ACE I/D polymorphism and dominant genetic model of AGT T704C polymorphism (Fig. 8). The results of the two polymorphisms showed that the blue line of the cumulative z-curve crossed the TSA monitoring boundary and the cumulative sample size was reached, indicating that no further studies were essential to confirm the associations.

## Discussion

In pregnant women with PE, downregulated renin-angiotensin system (RAS) activity is observed, resulting in increased vascular responsiveness to angiotensin II [4]. The increased plasm levels of angiotensin (AGT) and angiotensin converting enzyme (ACE) in PE subjects lead to the augmentation of angiotensin II [5, 54]; moreover, the pathophysiological effects of angiotensin II are enhanced by the upregulation of angiotensin II type 1 receptor (AT1R) [9], causing the dysregulation of blood pressure. Gene polymorphisms were reported to be associated with the abnormal expression of mRNA and protein [55, 56]. Our meta-analysis demonstrated that the polymorphisms of AGT T704C and ACE I/D were significantly associated with an increased risk of preeclampsia (PE) and weak associations of the AT1R A1166C polymorphism with PE were observed.

Previous meta-analyses indicated an increased PE risk with high heterogeneity of ACE I/D and AGT T704C polymorphisms, but no association was observed for the AT1R A1166C polymorphism [57–60]. However, the latest meta-analysis was performed in 2012, and in subsequent years, several studies conducted in different regions and ethnicities were published. An increased frequency of AT1R AC + CC genotypes in mild preeclamptic women was reported by Rahimi et al [9]. An interaction between the AGT T704C and ACE I/D polymorphisms and the risk of severe preeclampsia or the time onset of PE were observed [7, 8], but these were not analyzed in any former meta-analysis. Drawbacks in terms of high heterogeneity, slack inclusion criteria for subjects from different regions and ethnicities, the lack of evaluation of type 1 error and sample size on significant associations, the vague associations between these polymorphisms and the risk of severe PE, and different onset times of PE greatly aroused our interest. Therefore, we performed an updated meta-analysis with trial sequential analysis to consider the undiscussed above-mentioned issue. Regarding the AT1R A1166C polymorphism, significant associations in mixed race, early-onset, late-onset, and more than 200 patient sample size were discovered; however, only one study was analyzed in these subgroups, implying low representativeness of the AT1R A1166C polymorphism and further studies are essential.

In the overall analysis of the AGT T704C polymorphism, a 33% increased PE risk of CC + CT genotypes was observed. The 1.26-fold and 1.44-fold increased risk of PE in CT genotypes and CC genotypes, respectively, were also detected compared to TT genotypes. No heterogeneity in the genetic models and the positive results from the trial sequential analysis ensured the stability and reliability of our result. In the subgroup analysis stratified for geography, no significant association was detected; however, increased risks were observed in Caucasoid (the 1.30-fold and 1.28-fold increased risk of CC + CT genotype and CT genotype compared to TT genotype) and Mongoloid (the 60% increased of C allele in allelic genetic model; the 4.43-fold increased risk of DD genotype in recessive genetic model). In the severe PE degree subgroup analysis, no association was observed both in either severe or mild PE populations, possibly due to the small sample size, more studies are required. In the more than 200 patient sample size, increased risks were observed in the dominant, recessive, and heterozygote genetic models; however, the relatively small number of included studies in the subgroup indicated that these associations need to be interpreted with caution.

For the ACE I/D polymorphism, the D allele increased the risk of PE compared to I allele by 1.29-fold; moreover, the DD + DI, DD and DD genotypes increased risk by 17%, 52%, and 55% compared to II, DI + II, and II genotypes, respectively. Significant heterogeneity was observed in the overall analysis. We performed a Galbraith plot analysis to study potential heterogeneity analysis, and after excluding these studies [1, 18, 26, 32, 34, 45, 46], high heterogeneity was significant reduced. We did a comprehensive literature reviewed in these excluded studies; the mixed ethnicities, differences in geography, and patient sample size may be the reasons for the high heterogeneity. Therefore, a full subgroup analysis was conducted. In Asian populations including subjects from China, South Korea, Turkey, Iran, India, and Japan, the increased risk of PE in D allele (allelic genetic model), DD genotype (recessive genetic model), and DD genotype was 1.31-fold, 1.80-fold, and 1.53-fold, respectively. Regarding subjects from Europe (UK, Italy, Greece, and Norway), a 33% increased risk of PE in D allele (allelic genetic model) and a 68% increased risk of PE in DD genotypes (homozygote genetic model) were detected, appearing as though the Europeans had more risk of PE than did to Asians. In the subgroup analysis by ethnicity, increased risk of PE was only discovered in Caucasoid population, consistent with results of previous studies [57, 59, 61, 62]. We introduced PE degree and gestational week as subgroups to assess the potential relationships between the ACE I/D polymorphism and severe PE degree and onset time of PE. In the severe PE population, widely increased risks were observed, and we also detected a greater risk of PE than in the mild PE population. However, no significant association was detected for early-onset or late-onset of PE. For the patient sample subgroup analysis, increased risks were also observed.

There were several limitations in this meta-analysis. Firstly, language bias existed in our results; although no language limitation was set, only English and Chinese articles were included. Secondly, the sample size of included studies in the subgroup analysis of PE degree and onset time of PE were relatively small in some groups, implying that our results should be explained with caution. Finally, the potential influence of environment factors on genotype-PE associations is worthy of consideration.

Our results indicated that the AGT T704C and ACE I/D polymorphisms were associated with an increased risk of PE. Increased risks were also observed for the two polymorphisms in subgroups including Asians, Europeans, Caucasoid, and Mongoloid. Furthermore, an increased PE risk with the ACE I/D polymorphism in the severe PE population was also detected. Regarding the AT1R A1166C polymorphism, weak associations were observed and further studies are required.

## Electronic supplementary material

ESM 1 (DOCX 16 kb)

## Data Availability

The datasets generated during and/or analyzed during the current study are available from the corresponding author on reasonable request.
