# Supplementary material for: Three polymorphisms of renin-angiotensin system and preeclampsia risk
Source: J Assist Reprod Genet. 2020 Nov 23;37(12):3121–42. doi: 10.1007/s10815-020-01971-8 (PMC7714824; doi:10.1007/s10815-020-01971-8)
Supplement: Supplementary file 1 — (DOCX 16 kb) [file 10815_2020_1971_MOESM1_ESM.docx]

| Table S2. Methodological quality assessment for included studies. | | | | | | | |  |  |
| --- | --- | --- | --- | --- | --- | --- | --- | --- | --- |
| Criteria | | | | | | | | | Score |
| 1. Representativeness of cases | | | | | | | | |  |
|  | Preelampsia diagnosed according to acknowledged criteria. | | | | | | | | 2 |
|  | Mentioned the diagnosed criteria but not specifically described. | | | | | | | | 1 |
|  | Not Mentioned. | | | | | | | | 0 |
| 2. Source of controls | | | | | | | | |  |
|  | Population or community based. | | | | | | | | 3 |
|  | Hospital-based coronary artery disease free controls. | | | | | | | | 2 |
|  | Congenital heart disease free controls with related diseases. | | | | | | | | 1 |
| 3. Sample size | | | | | | | | |  |
|  | ≥300 | | | | | | | | 2 |
|  | <300 | | | | | | | | 1 |
| 4. Quality control of genotyping methods. | | | | | | | | |  |
|  | Repetition of partial/total tested samples with a different method. | | | | | | | | 2 |
|  | Repetition of partial/total tested samples with a same method. | | | | | | | | 1 |
|  | Not described. | | | | | | | | 0 |
| 5. Hardy-Weinberg Equilibrium (HWE) | | | | | | | | |  |
|  | Hardy-Weinberg Equilibrium in control populations. | | | | | | | | 1 |
|  | Hardy-Weinberg Disequilibrium in control populations. | | | | | | | | 0 |
|  |  |  |  |  |  |  |  |  |  |
